# Prevalence and Associated Factors of Anemia Among Pregnant Women Attending Antenatal Care at Mizan Aman General Hospital, Bench Maji Zone, Southwest, Ethiopia

**DOI:** 10.1155/anem/2655891

**Published:** 2024-11-14

**Authors:** Kaleab Tesfaye Tegegne, Seblework Abeje, Eleni Tesfaye Tegegne, Mekibib Kassa Tessema, Tadele Kassahun Wudu

**Affiliations:** ^1^Department of Public Health, College of Health Science, Debark University, Debark, Ethiopia; ^2^Department of Public Health, College of Health Science, Mizan Tepi University, Mizan Tepi, Ethiopia; ^3^School of Nursing, College of Medicine and Health Science, University of Gondar, Gondar, Ethiopia; ^4^Leshmania Research and Treatment Center, University of Gondar, Gondar, Ethiopia; ^5^Department of Statistics, Debark University, Debark, Ethiopia

**Keywords:** anemia, associated factors, Ethiopia, pregnant women, prevalence

## Abstract

**Background:** Anemia is one of the most common nutritional deficiency disorders affecting pregnant women; its prevalence in developed countries is 14% and in developing countries 51%. It is therefore important to understand the prevalence and associated factors of anemia in our study area. This will encourage antenatal caregivers to identify and treat anemia early in pregnancy.

**Objective:** Therefore, the study's goal was to determine the prevalence of anemia and its contributing factors among pregnant women receiving prenatal care.

**Methods:** Between April 2021 and May 2021, 295 pregnant women attending prenatal care participated in a cross-sectional facility-based study. Epidata software was used to enter the data, which were then exported to SPSS software for Windows version 23 for analysis. To determine the factors contributing to anemia in pregnant women, descriptive statistics collected with the study were performed together with bivariable logistic regression and the log-binomial model.

**Results:** Among the 295 study participants, 24.7% were anemic. Out of these, most were mild types 78.1%. Illiterate pregnant women (ARR 2.89; 95% CI: 1.76–6.43, *p* value = 0.037), with no iron-containing food intake per day (ARR 1.74; 95% CI: 1.59–1.95, *p* value = 0.01), and infected with malaria (ARR 1.58; 95% CI: 1.76–2.53, *p* value = 0.03) had higher odds of being anemic, compared to their counterpart. Gestational age of the first (ARR 0.21; 95% CI: 0.03–0.98, *p* value = 0.01), and second (ARR 0.8; 95% CI: 0.43–0.96, *p* value = 0.013) trimester has lower odds of being anemic compared to their counterpart.

**Conclusion:** Anemia in pregnant women is found to be a moderate public health issue in the research location. It is strongly and independently impacted by malaria infection and iron-containing meal consumption. Reducing the prevalence of anemia is made possible by improved iron-containing meal consumption. In addition, it is strongly advised that pregnant women receive education and should take iron supplements during pregnancy visits.

## 1. Introduction

The World Health Organization defined anemia during pregnancy as a hemoglobin level of < 11 g/dL [1]. Anemia is a worldwide public health issue that significantly affects expectant mothers [2]. Iron deficiency anemia (IDA) has reached epidemic proportions in underdeveloped countries and has become a serious global public health problem, affecting particularly 0–5-year-old children and young women of reproductive age, especially during pregnancy. A life-threatening loss of red blood cells, muscle function, and the ability to produce energy can result from an iron deficit [3].

Anemia affects 20% of pregnant women, with iron, folic acid, or both deficiencies accounting for the majority of cases. Pregnant women's use of iron and folic acid is a contentious topic, with different nations adhering to different principles [4]. It is recommended that all expectant mothers undergo screening for IDA and receive iron supplementation if necessary. The UK recommendations also state that women with nonanemic iron deficiency should be treated with 65 mg of iron per day [5]. Pregnancy increases the need for iron compared to nonpregnancy. Iron requirements increase significantly after the first trimester when they are lowered due to the lack of menstruation; for a 55-kg woman, this amounts to approximately 1000 mg [6].

Smaller daily iron doses (30 mg/day) are used in the United States as a main preventive measure for iron supplementation; nevertheless, therapeutic doses of up to 120 mg elemental iron daily are advised for treating anemia [7]. The volume of plasma gradually rises during a typical pregnancy. The majority of this 50% increase happens by 34 weeks of gestation and is correlated with the baby's birth weight. There is a decrease in the hemoglobin concentration, hematocrit, and red blood cell count because the expansion of plasma volume is more than the rise in red blood cell mass [8].

Anemia in pregnancy leads to premature births, low birth weight, fetal impairment, and infant deaths. It also reduces the productivity of women [4]. In Ethiopia, previous studies have linked anemia to factors such as child age, child nutritional status, parents' educational level, and wealth index [9].

Anemia is a prevalent nutritional deficiency illness that affects pregnant women. Its incidence varies between 65% and 75% in India, 51% in underdeveloped nations, and 14% in affluent countries. Anemia is responsible for around 80% of maternal fatalities in South East Asia [10]. According to estimates from the World Health Organization, over 50% of pregnant women and over 40% of nonpregnant women in underdeveloped nations are impacted [4].

Compared to neonates of nonanemic maternal women, the odds of preterm delivery, perinatal death, and early neonatal mortality were also observed to be greater in the offspring of anemic maternal women [11]. In Ethiopia, the combined prevalence of anemia among expectant mothers was 26.4% [12].

Hemoglobin levels below 110 g/L, or anemia, were prevalent, accounting for 40.6% of births, 64.7% of second prenatal visits, and 68.3% of first prenatal visits [13]. In 2016, anemia during pregnancy affected 40.05% of pregnant women worldwide, with Southeast Asia having the greatest frequency (48.15%). Due to the large prevalence of anemia, any negative effects on the mother and fetus that result from anemia during pregnancy would have a significant influence on public health [14].

Pregnancy-related anemia was predicted by the women's employment and level of literacy [15]. Previous studies in Southern Ethiopia have identified rural residence, previous history of heavy menstrual blood flow, age of mother, parasitic infection, food taboo (aversion), and drinking tea/coffee instantly after meal were predictors of anemia among pregnant women [16]. The strongest correlation between low serum retinol and mild anemia was found, while the degree of hookworm infection and *P. vivax* malaria were more powerful indicators of moderate to severe anemia [17].

Anemia cannot be recovered from quickly when there is malaria infection because it results in bone marrow dyserythropoiesis and hemolysis of both infected and uninfected erythrocytes [18]. The contextual factors contributing to anemia among pregnant women are different There are now a lot of pertinent studies, but most of them concentrate on anemia that occurs only once during pregnancy or anemia at a specific point in the pregnancy; anemia that occurs throughout the entire pregnancy is not well-researched. The research area lacks sufficient information regarding the factors that contribute to anemia in pregnant women. This study's objectives were to ascertain the prevalence of anemia and associated factors in the study area, to compare the findings with findings obtained nationwide, and to offer any appropriate advice. It is therefore important to understand the prevalence and associated factors of anemia at Mizan Aman General Hospital. It is also used to establish baseline data for further research due to the scarcity of data at Mizan Aman General Hospital.

## 2. Materials and Methods

### 2.1. Study Area and Period

The study was carried out at the Mizan Aman General Hospital in the Bench Maji district of the Southern nation, 566 km from Addis Ababa. It offers antenatal care, family planning, outpatient and inpatient care, and it has four departments: surgery, obstetrics and gynecology, pediatrics, and medicine. It serves a population of 2,000,000, with a total annual patient flow of 34,314 in 2021 and 3600 pregnant women receiving ANC visits in that year. The study was carried out from April to May 2021.

### 2.2. Study Design

A facility-based cross-sectional study was conducted to gauge the prevalence of anemia and related factors among pregnant mothers.

### 2.3. Source Population

It includes all pregnant mothers who are present for their ANC follow-up appointment at Mizan Aman General Hospital.

### 2.4. Inclusion Criteria

The inclusion criteria include pregnant women attending at least their first ANC appointment and pregnant women continuing to be permanent residents (at least 6 months).

### 2.5. Exclusion Criteria

The exclusion criteria include any pregnant woman admitted to an inpatient ward.

### 2.6. Sample Size Determination and Sampling Technique

By applying a single population proportion calculation and using a 22% prevalence of anema, the sample size was estimated [19], using a confidence interval of 95% and a margin of error of 5%. Given that *z*_*a*/2_ = 1.96,

(1) $$\begin{array}{c} n={\left(z_{a/2}\right)}^{2}p\frac{\left(1-p\right)}{d^{2}}\\ n={\left(1.96\right)}^{2}\times 0.22\times \frac{\left(1-0.22\right)}{\left(0.05\right)}\\ n=\frac{.0.672}{.0025}\\ n=269.1\\ n=269. \end{array}$$

By adding a nonresponse rate of 10%, the final sample size was 295.

Where *Z* = standard score corresponding to 95% confidence Internali(CI).

*d* = margin of error

*n* = sample size

*p* = prevalence of anema

During the data-collecting period, study subjects were sequentially recruited using the systematic random sampling technique when they arrived at the hospital's prenatal care clinic. *N* = 300 pregnant women came for ANC from April to May 2021.*K* = *N*/*n* = 300/295 = 1.01

*K* ^th^ = 2 so every 2nd pregnant women were selected

### 2.7. Variables

The dependent variable Anemia: Hemoglobin below 11 gm/dL.

### 2.8. Independent Variables' Includion

  Sociodemographic characteristics: age, occupation, marital status, income religion, educational status, and residence.
  Gynecologic and obstetric factors: abortion, parity, and menstrual cycle (the cycle is not the same for everyone. Menstrual bleeding might happen every 21–35 days), family planning use, gestational age)
  Other factors: feeding habit**s,** malarial infection, and iron supplementation.

An anthropometric assessment, laboratory analysis of stool and blood samples, and interviews with pregnant women who receive antenatal care at the Mizan Aman Health Center were used to compile the data for the questionnaire, which also included information on sociodemographic, clinical, and reproductive, nutrition and lifestyle, and knowledge and health service–related factors.

Iron-containing food intake per day was measured using an iron dietary intake questionnaire based on a food frequency assessment. To uncover the dietary pattern of anemia, the Food Frequency Questionnaire (FFQ) was considered a great tool for assessing dietary habits for this study. As a result, for the current study, a predesigned FFQ was standardized and changed according to the diet of local individuals, with adjustments to local food items. Specifically, accessible grains, cereals, and fruits were included to monitor the nutrient consumption of pregnant women receiving antenatal care at Mizan Aman General Hospital.

To measure a group of pregnant women attending antenatal care through nutritional intake, the 24-h recall method is well-established and efficient. Thus, this strategy was used in filling out the questionnaire for our review, where the responder recalls when and how much food was ingested. Participants were asked to fill out the FFQ based on the consumption pattern of their diets, and frequency of consumption of various food items which included cereals, pulses, vegetables, fruits, milk and milk products, meat, fish and poultry, cooking oil, ghee, sweets, nuts, and dry fruits, fruit juice, and junk foodstuffs.

### 2.9. Data Collection Procedure

The literature was reviewed to design the questionnaire [20–26]. When assessing a questionnaire's internal consistency, Cronbach's alpha is typically employed. To proceed with this research, the study instruments must have reliability of 0.7 or above [27]. After transforming the responses into constructs in SPSS and running the test, it is found that Cronbach's alpha value is 0.87 which means that all the instruments used in this research are reliable enough. For the questionnaire/data collection content, researchers reviewed the questions after they had been written in English, translated into the target language, and then back into English. Data collection involved inputs from every investigator. The questionnaire's section on dietary diversity (DD) was taken from a common instrument [28].

### 2.10. Blood Sample Collection and Analysis in the Lab

Hemoglobin was determined using HemoCueÒ, and red blood cells (RBC) shape was identified microscopically [29]. This Hgb determination was performed on 295 pregnant mothers who regularly get antenatal follow-ups at Mizan Aman General Hospital.

### 2.11. Data Analysis and Processing

Epidata software was used to enter the data, which was then exported to SPSS for Windows version 23 for analysis. To determine the factors connected to anemia in pregnant women, descriptive statistics were calculated together with both bivariable logistic regression and the log-binomial model. We have used a binary logistic regression model. The Hosmer–Lemeshow test is a statistical test for goodness of fit for binary logistic regression but the *p* value for the Hosmer–Lemeshow test was 0.02 which is less than 0.05 so that the model is not fit and we have used the log-binomial model. In the bivariable logistic regression, we have calculated the crude odd ratio with *p* values. We have entered the variables with a *p* value less than 0.05 in bivariable logistic regression to the log-binomial model and adjusted risk ratios (ARRs) with *p* values being calculated in the log-binomial model.

### 2.12. Data Quality Control

Daily checks on the completed questionnaires' completion were done by the lead investigator to ensure the quality of the data during the data collection process.

### 2.13. Ethical Considerations

The Institutional Research Ethics Review Committee at Mizan Tepi University drafted an official letter of consent outlining the study's goals for the hospital before the study began. The acquired data were handled in confidence and were not identified by names. The patient was not harmed physically or psychologically by the trial. The responders were handled with respect, and through anonymity, their right to privacy and confidentiality was upheld. Written informed consent was received.

## 3. Results

According to our study result, 82.7% of the respondents were multigravidae and 17.3% were primigravidae. In addition, 50.8% of the respondents were taking iron regularly during the current pregnancy. According to our study result, 44.4% of the respondents were illiterate, 92% were married, 56% were housewives, and 56.6% came from urban areas (Table 1).

**Figure 1:**
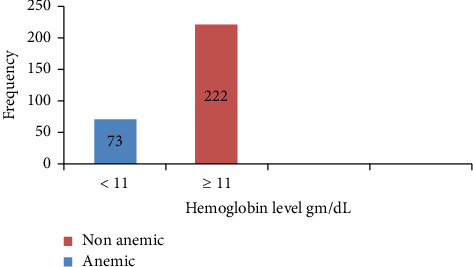
Prevalences of anemia among pregnant women attending antenatal care at Mizan Aman General Hospital, 2021.

Anemia: 24.7% (73) of the respondents were anemic (Figure 1).

**Figure 2:**
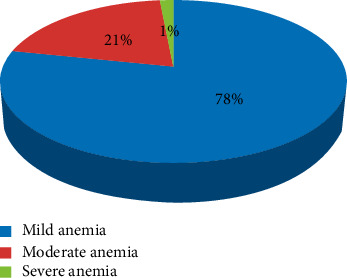
Severity of anemia among pregnant women attending ANC follow-up at Mizan Aman General Hospital, 2021.

Severity of anemia: 78% of the respondents were mild anemic (10–10.9 gm/dL) (Figure 2).

Sociodemographic factors: A pregnant woman who was illiterate (ARR 2.89; 95% CI: 1.76–6.43, *p* value = 0.037), with low monthly family incomes (ARR 1.76; 95% CI: 1.32–4.54, *p* value = 0.001), had higher odds of being anemic with compared to their counterparts (Table 2).

### 3.1. Obstetric, Gynecologic, and Nutritional Factors

Those with a history of malaria infection (ARR 1.58; 95% CI: 1.76–2.53, *p* value = 0.03), did not consume any iron-rich foods daily (ARR 1.74; 95% CI: 1.59–1.95, *p* value = 0.01), a history of abortion (ARR 3.72; 95% CI: 1.76–4.97, *p* value = 0.014), first- (ARR 0.21; 95% CI: 0.03–0.98, *p* value = 0.01) and second-trimester gestational levels (ARR 0.8; 95% CI: 0.43–0.96, *p* value = 0.013), and a menstrual cycle (menstrual history < 21 days) (ARR 2.42; 95% CI: 1.48–15.21, *p* value = 0.021) had higher odds of being anemic than their counterparts (Table 3).

## 4. Discussion

In the study area, 24.7% of pregnant women were anemic. Concerning the WHO cutoff points [23], the magnitude indicates moderate public health significance of anemia in the study area. This prevalence was comparable to the national prevalence of anemia among women of reproductive age in previous studies [19, 30] but higher compared to another study in Ethiopia which is 56.8% [31] but lower compared to reports from developing countries [10]. The possible reason for the observed difference may have resulted from differences in sample size and sociodemographic characteristics. In this study, mild anemia was the highest in magnitude and second was moderate anemia. This is consistent with reports from the different parts of the country [30, 32–34]. The mean Hgb concentration in the present study was 12.67 g/dL. This is consistent with the study report [35].

Pregnancy is the most iron-demanding period in a woman's life. Consequently, pregnant women are advised to eat a more iron-containing diet than usual. However, this was not the case in the study area. The level of iron-containing diet among pregnant women significantly determined their anemic level in the study. This is in agreement with the national study in Liberia among the reproductive age group [19]. A possible explanation might be the presence of food taboos during pregnancy. In some regions of Ethiopia, pregnant women were not allowed to eat fruits, vegetables, or fatty foods such as meat or dairy items. [36].

This study demonstrated that mothers who have low monthly family incomes were more likely to be anemic as compared to those with high monthly family incomes. This is in agreement with the study conducted at Eastern Zone of Tigray, Ethiopia [34]. Moreover, in this study, 71.7% of study participants were from urban areas, suggesting that they were food net buyers. As the income is low, the expenditure on food becomes low. Besides, due to food price inflation, the purchasing power of income is low. So, low-income groups did not get adequate nutrition, and thereby low–family income groups were at risk of anemia.

The pregnant women at the first- and second-trimester gestational periods were less likely to develop maternal anemia compared to those in the third trimester in this study. This might be due to an increase in hemodilution as a result of an increase in estrogen levels toward the end of gestational age [37]. The increase in anemia prevalence in the third trimester ascertained in this study was similar to what was reported in other studies [37].

In this study, the supplementation of iron supplements as nutrition therapy during the current pregnancy period did not significantly reduce the prevalence of anemia as compared to those who did not take these supplements. The possible reason may be that, in anemic pregnant women, these nutritional supplements were more likely to be prescribed as an intervention for the management of anemia during their ANC visit. This needs to be further studied to explicitly explain how effective the current WHO nutritional supplementation recommendation program is being implemented in the country and study area for the prevention and control of anemia in pregnant women.

In addition, in the study illiterate and those with a history of abortion had higher odds for anemia as compared to their counterparts. This study was similar to what was reported in other studies [38–40]. In this study, anemic cases were two times more likely to have a history of short menstrual cycle of less than 21 days before the index pregnancy, a finding similar to what was reported in other studies [41].

### 4.1. Limitations of the Study

There could have been recall and/or social desirability bias while subjects were requested to give dietary information and monthly income. Given the high prevalence of submicroscopic malaria infection and self-reported data on history of malaria infection, this study could be subjected to recall bias, and genetic problems and other potential factors linked to anemia were not taken into consideration in this investigation.

The small sample size further restricts the applicability of the findings to a larger population due to reduced statistical power and reliability. Stool concentration technique and parasite density/stool examination were not done in this study, so we could not assess the impact of parasite load on the severity of anemia.

## 5. Conclusion

Anemia among pregnant women was found to be a moderate public health problem in the study area. In addition, malaria infection, a history of a previous abortion, a third-trimester gestational period, low monthly family incomes, illiteracy, a history of excessive menstrual bleeding, and no iron-containing meal consumption significantly and independently affected the anemia of pregnant women. Using family planning methods and improved iron-containing meal consumption contributes to decreasing the prevalence of anemia, which should be advocated more as well, and educating pregnant women on this practice is highly recommended.

## Figures and Tables

**Table 1:** Sociodemographic characteristics of pregnant women attending ANC visits at Mizan Aman General Hospital 2021 (*n* = 295).

Variable	Detail	Frequency (*n*)	Percentage (%)
Age (years)	< 18	12	4.07
	18–35	224	75.9
	35–49	59	20.3

Residence	Urban	128	56.6
	Rural	167	43.4

Religion	Protestant	140	47.5
	Orthodox	58	32.9
	Muslim	97	19.7

Ethnicity	Bench	136	46.2
	Kefa	67	22.7
	Amhara	60	20.3
	Others	32	10.8

Educational statues	Illiterate	131	44.4
	Can read and write	70	23.7
	Primary	27	9.21
	Secondary	31	10.49
	College and above	36	12.2

Marital status	Single	11	3.7
	Married	271	92
	Widowed	8	2.6
	Divorced	5	1.7

Occupation	Housewife	168	56.9
	Merchant	52	17.6
	Civil servant	63	21.4
	Others	12	4.1

Monthly income (ETBIRR)	< 500	97	32.9
	500–1000	132	44.75
	> 1000	66	22.35

Abbreviation: ETBIRR = Ethiopia Birr.

**Table 2:** Anemia and sociodemographic factors associated with it among pregnant women attending ANC follow-up in Mizan Aman General Hospital, Mizan Aman, Ethiopia, 2021, (*n* = 295).

Variable		Anemia		Bivariate analysis COR 95% CI	*p* value	Log binomial regression Adjusted ARR 95% CI	*p* value
		Yes	No				
Age	< 18	3 (25%)	9 (75%)	1.00		1.00	
	18–35	54 (24.1%)	170 (75.9%)	1.049 (0.274–4.015)	0.07	1.2 (0.3–3.8)	0.056
	35–49	16 (27.1%)	43 (72.9%)	0.896 (0.215–3.732)	0.08	0.9 (0.34–2.89)	0.054

Residence	Urban	33 (25.8%)	95 (74.2%)	1.00			
	Rural	40 (23.9)	127 (76.1%)	1.103 (0.647–1.88)	0.06	1.4 (0.72–1.98)	0.058

Educational statues	College and above	5 (13.9)	31 (86.1%)	1.00			
	Secondary	4 (12.9%)	27 (87.1%)	0.919 (0.22–3.77)	0.09	1.12 (0.32–4.56)	0.062
	Primary	12 (44.4%)	15 (55.6%)	4.96 (1.48–16.6)	0.06	3.97 (1.97–19.12)	0.052
	Read and write	21 (30%)	49 (70%)	2.657 (0.91–7.78)	0.07	3.87 (0.78–8.65)	0.072
	Illiterate	31 (23.6%)	100 (76.4%)	1.922 (1.69–5.37)	0.032⁣^∗^	2.89 (1.76–6.43)	0.037⁣^∗^

Marital status	Single	3 (27.3%)	8 (72.7)	1.00			
	Married	67 (24.7%	174 (75.3%)	0.974 (0.717–2.2025)	0.055	1.6 (0.65–1.89)	0.082
	Widowed	3 (37.5%)	5 (62.5%)	1.014 (1.498–2.422)	0.054	1.7 (1.98–3.54)	0.071

Monthly income (Ethiopian Birr)	< 300	3 (27.3%)	49 (70%)	1.89 (1.46–5.68)	0.023⁣^∗^	1.76 (1.32–4.54)	0.001⁣^∗^
	301–600	21 (30%)	31 (86.1%)	4.5 (0.43–5.4)	0.054	5.1 (0.99–7.12)	0.087
	601–1000	33 (25.8%)	37 (72.5%)	3.38 (0.98–6.8)	0.093	2.65 (0.78–5.34)	0.056
	> 1001	9 (75%)	4 (13.7%)	1.00			

Abbreviations: COR, crude odd ratio; ARR, adjusted relative risk; CI, confidence interval.

^∗^indicates a statistically significant result that is p value less than 0.05.

**Table 3:** Relationship of anemia to past obstetric, gynecologic history, and nutritional history in pregnant women attending ANC follow-up in Mizan Aman General Hospital 2021 (*n* = 295).

Variable	Details	Anemia		Bivariate analysis COR 95% CI	*p* value	Log binomial regression adjusted ARR 95% CI	*p* value
		Yes	No				
Gravidae	Primigravidae	14 (27.5%)	37 (72.5%)	1.00			
	Multigravidae	59 (24.2)	185 (75.8%)	1.186 (1.66–2.26)	0.052	1.65 (1.54–2.12)	0.064

Abortion	No	50 (19.8%)	202 (90.1%)	1.00			
	Yes	23 (53.5%)	20 (46.5%)	4.646 (1.822–2.098)	0.021⁣^∗^	3.72 (1.76–4.97)	0.014⁣^∗^

Family planning	Yes	48 (24.6%)	147 (75.4%)	1.00			
	No	25 (25%)	75 (75%)	0.979 (0.561–1.771)	0.053	0.87 (0.43–1.57)	0.08

Menstrual history (menstrual cycle)	> 35 days	4 (13.7%)	25 (86.3%)	1.00			
	21–35 days	63 (25.5%)	184 (74.5%)	2.14 (0.72–6.39)	0.061	1.67 (0.47–5.89)	0.067
	< 21 days	6 (31.5%)	13 (68.5%)	2.88 (1.69–12.07)	0.01⁣^∗^	2.42 (1.48–15.21)	0.021⁣^∗^

Gestational age	> 27 third	36 (25.5%)	101 (74.5%)	1.00			
	14–26 s	36 (24.3%)	112 (75.7%)	0.9 (0.532–0.954)	0.023⁣^∗^	0.8 (0.43–0.96)	0.013⁣^∗^
	≤ 13 first	1 (10%)	90 (90%)	0.31 (0.04–0.55)	0.03⁣^∗^	0.21 (0.03–0.98)	0.01⁣^∗^

History of malaria attack	No	48 (22.1%)	169 (77.9%)	1.00			
	Yes	25 (32%)	53 (68%)	1.861 (1.936–2.95)	0.04⁣^∗^	1.58 (1.76–2.53)	0.03⁣^∗^

Tea and coffee intake per day	No	3 (3.8%)	77 (96.2%)	1.00			
	Yes	70 (32.5%)	145 (67.5%)	12.391 (3.777–40.65	0.08	11.08 (2.88–33.08)	0.076

Iron-containing food intake per day	Yes	40 (34.1%)	131 (65.9%)	1.00			
	No	33 (26.6%)	91 (73.4%)	1.842 (1.494–1.835)	0.02	1.74 (1.59–1.95)	0.01⁣^∗^

Iron supplementation	Yes	39 (26%)	111 (%)	1.00			
	No	34 (23.4%)	111 (%)	1.147 (0.675–1.948	0.054	1.07 (0.78–1.56	0.056

Abbreviations: ARR = adjusted relative risk, CI = confidence interval, and COR = crude odd ratio.

^∗^indicates a statistically significant result of p value <0.05 for variables that have a statistically significant association with anemia.

## Data Availability

The data used to support the findings of this study are available from the corresponding author upon reasonable request.

## Nomenclature

ANC: Antenatal care
ARR: Adjusted relative risk
CI: Confidence interval
COR: Crude odd ratio
DD: Dietary diversity
ETBIRR: Ethiopian Birr
FFQ: Food Frequency Questionnaire
Hgb: Hemoglobin level
RBC: Red blood cell
UN: United Nations
WHO: World Health Organization
